# Dataset on raindrop size distribution, raindrop fall velocity and precipitation data measured by disdrometers and rain gauges over Peruvian central Andes (12.0°S)

**DOI:** 10.1016/j.dib.2020.105215

**Published:** 2020-01-31

**Authors:** Jairo M. Valdivia, Kevin Contreras, Daniel Martinez-Castro, Elver Villalobos-Puma, Luis F. Suarez-Salas, Yamina Silva

**Affiliations:** Instituto Geofísico del Perú, Peru

**Keywords:** DSD, Disdrometer, In situ atmospheric observations, Huancayo observatory

## Abstract

This dataset includes data obtained at the Atmospheric Microphysics and Radiation Laboratory (LAMAR) of the Huancayo Observatory (12.04° S, 75.32° W, 3313 m ASL). Two Parsivel2 and two tipping bucket rain gauges are used in this dataset which are operating together since 2018. Data is given in NetCDF format, including two types of files, one NetCDF for precipitation totals and another which contains Parsivel2 data. This data set was collected in the complex topography conditions of the tropical Andes, and its potential use is to study the microphysics of orographic rainfall, atmospheric models and rainfall estimation algorithms.

**Table:** Specifications Table

Subject	Atmospheric Science, Meteorology
Specific subject area	Rainfall microphysics, raindrop sizes and fall velocities
Type of data	Numerical matrix (NetCDF) Table
How data were acquired	Laser-Optical Disdrometer, OTT Parsivel2 Tipping-bucket rain gauge, Texas Electronics, Model No.: TE525 Tipping-bucket rain gauge, Hyquest Solutions, Model No.: CS700
Data format	Raw
Parameters for data collection	The instruments were installed in an open field between 1 and 2 m of distance above the ground.
Description of data collection	The instruments are automatic and operates full time. The data collection only stops when there are electrical problems in the observatory.
Data source location	Huancayo Observatory - Instituto Geofísico del Perú Mantaro river valley, Junín Perú Latitude and longitude: 12°02′18″S, 75°19′22″W
Data accessibility	Raindrop size distribution and precipitation over Peruvian central Andes (12.0°S) 10.5281/zenodo.3497564 : https://scah.igp.gob.pe/sites/datos/PP_prods/

**Table:** 

**Value of the Data** • It is the only in situ dataset of drop size distribution over the tropical central Andes. Precipitation measurements over the Andes are scarce, particularly at high time resolution. • These data can be used by scientists and academic community interested in studying rain microstructure, atmospheric models and rainfall estimation algorithms. • This data is useful to evaluate the performance of high resolution atmospheric models, and to evaluate quantitative precipitation estimation algorithms.

## 1 Data

**Fig. 1:**
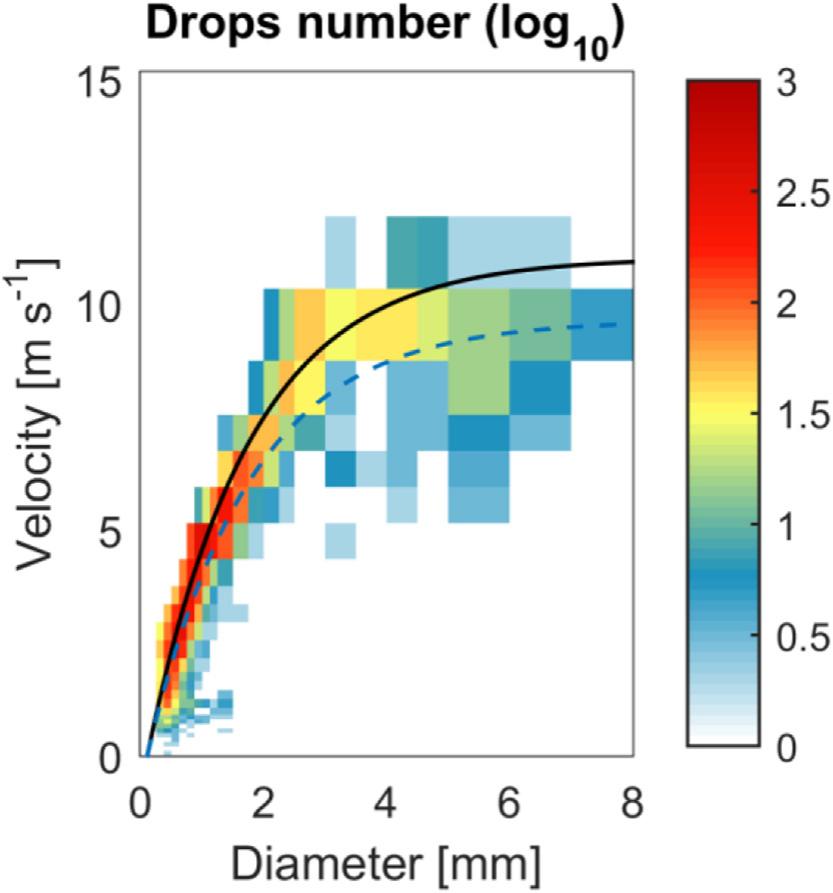
Example of Parsivel2 raw output. Drops number in a matrix of diameter versus velocity. The black line indicates the theoretical relationship based on [4] data and the blue dashed line same as black but the air dependence is corrected for 3300 m ASL following [5,6].

All the data was automatically collected by the disdrometers and rain gauges. Two optical disdrometers Parsivel2 and two tipping-bucket rain gauges are used. The Parsivel2, manufactured by OTT, measured the size and velocity of hydrometeors [1]. The sampling output interval is 1 min, it is recorded as a plain text and then are converted to NetCDF. Parsivel2 outputs the drops data in a 32 × 32 matrix of size versus velocity (see Fig. 1). Additionally, rainfall rate, radar reflectivity and others rain parameters are computed from drop size distribution by the Parsivel2 internal software. Each Parsivel2's NetCDF has the structure as is shown in Table 1. The time in this dataset is in the Matlab format (i.e. number of days since 01-01-0000 UTC), the bin class diameter D and bin class velocity vel of the raw data matrix are in mm and $ms^{-1}$, respectively. The rain rate and the radar reflectivity factor are $mmh^{-1}$ and dBZ (or $10\times \log_{10}\left[mm^{6}m^{-3}\right]$), respectively. The SYNOP4680 and SYNOP4677 are weather code according to tables 4680 and 4677 of World Meteorological Organization (WMO). The Drop density Nd of the drop size distribution (i.e. number of drops per meter cube and class spread) is in $\log m^{-3}mm^{-1}$. The bin class spread diameter dspread and bin class spread velocity vspread are in mm and $ms^{-1}$, respectively.

**Table 1:** Parsivel2's NetCDF structure. (view from the terminal and ncdump tool).

jvaldivia@IGPmaster2:/data/datos/LAMAR/PP_prods/PSV_nc/PSVa > ncdump -h PARSIVEL_20190301.nc netcdf PARSIVEL_20190301 { dimensions: time = UNLIMITED; //(1440 currently) D = 32; vel = 32; variables: double time(time); time:long_name = “Days since 01.01.0000 00:00 UTC (MatLab format)”; time:units = “days”; float D(D); D:long_name = “Drop diameter bin centered”; D:units = “mm”; float vel(vel); vel:long_name = “Drop velocity”; vel:units = “m/s”; float RR(time); RR:long_name = “Rain Rate”; RR:units = “mm/h”; float Z(time); Z:long_name = “Radar Reflectivity Factor Z”; Z:units = “dBZ”; float SYNOP4680(time); SYNOP4680:long_name = “Weather code acc. to SYNOP; Table 4680”; SYNOP4680:units = “ ”; float SYNOP4677(time); SYNOP4677:long_name = “Weather code acc. to SYNOP; Table 4677”; SYNOP4677:units = “ ”; float Nd(time, D); Nd:long_name = “Drop density”; Nd:units = “log 1/mˆ3 mm”; float raw(time, D, vel); raw:long_name = “Raw data”; raw:units = “1”; float dspread(D); dspread:long_name = “Bin class spread diameter”; dspread:units = “mm”; float vspread(vel); vspread:long_name = “Bin class spread velocity”; vspread:units = “mm”; }

The total rainfall registered by all the instruments (i.e., both Parsivel2 and both rain gauges) are given in another NetCDF which his structure is shown in Table 2. In the same way, the time in these files is in the Matlab format. The precipitation measured by the rain gauges, pp_pluv_meteo and pp_pluv_exper, are in mm (which represent the precipitation measured in each minute). The precipitation measured by both Parisvel2, pp_parisvel2_a and pp_parsivel2_b, are in mm as well.

**Table 2:** Total rainfall NetCDF structure.

jvaldivia@IGPmaster2:/data/datos/LAMAR/PP_prods/PP_dia > ncdump -h PP20180805.nc netcdf PP20180805 { dimensions: time = 1440; variables: double time(time); time:units = “formato numerico”; double pp_pluv_meteo(time); pp_pluv_meteo:long_name = “Total de precipitacion - Pluviometro Meteorologico”; pp_pluv_meteo:units = “mm”; double pp_pluv_exper(time); pp_pluv_exper:long_name = “Total de precipitacion - Pluviometro Experimental”; pp_pluv_exper:units = “mm”; double pp_parsivel2_a(time); pp_parsivel2_a:long_name = “Total de precipitacion - Parsivel2 - a”; pp_parsivel2_a:units = “mm”; double pp_parsivel2_b(time); pp_parsivel2_b:long_name = “Total de precipitacion - Parsivel2 - b”; pp_parsivel2_b:units = “mm”; }

All the NetCDF in this database contains a day of data.

## 2 Experimental design, materials, and methods

Both Parsivel2 and both rain gauges used in this database are installed in the Huancayo Observatory of the Instituto Geofísico del Perú (12.04° S, 75.32° W, 3313 m ASL), located in the Mantaro valley, in the central Andes of Peru at 12 km from Huancayo city, between the western Andean Cordillera and the Huaytapallana Cordillera to the east. Annual rainfall climatology at this location is 700 mm and it is characterized as being dry in winter (austral) and rainy in summer. According to Köppen-Geimer classification [2], Mantaro valley has an arid cold steppe climate.

The retrieval of drop size distribution (DSD) of Parsivel2 has been evaluated by Ref. [3]. The raw output is the number of drops at the $i^{th}$ size and $j^{th}$ velocity bin ($C_{i,j}$), and DSD is calculated as

$$\text{N}\left(\text{D}\right)=\frac{1}{\text{Time}}\;\overset{\text{n}}{\underset{\text{i}=1}{\sum }}\overset{\text{m}}{\underset{\text{j}=1}{\sum }}\frac{C_{i,j}}{v_{t}Area\left(D_{i}\right)\text{Δ}D_{i}}\;,$$

where, $\text{Δ}D_{i}$ is the width of the $i^{th}$ size bin; and n and m are the size and velocity bins, respectively, and both are equal to 32. $Area\left(D_{i}\right)$, the effective sampling area is calculating considering partially detected drops across Parsivel2's laser sheet and is equal to $180mm\times \left(130mm–D_{i}/2\right)$. $v_{t}$ is the measured raindrop fall speed at the $j^{th}$ velocity bin. Fig. 1 shows how the Parsivel2 raw output data looks like. A MATLAB toolbox to manipulate the Parsivel2 data is available at https://github.com/JValdivia23/parsivel2 [7].
